# A Terrain Perception Method for Quadruped Robots Based on Acoustic Signal Fusion

**DOI:** 10.3390/s26020594

**Published:** 2026-01-15

**Authors:** Meng Hong, Nian Wang, Xingyu Liu, Chao Huang, Ganchang Li, Zijian Li, Shuai Shu, Ruixuan Chen, Jincheng Sheng, Zhongren Wang, Sijia Guan, Min Guo

**Affiliations:** 1 School of Mechanical and Electronic Engineering, Wuhan University of Technology, Wuhan 430070, China; hongmeng@whut.edu.cn (M.H.); nwang@whut.edu.cn (N.W.); liuxingyu69@whut.edu.cn (X.L.); 297179@whut.edu.cn (C.H.); 360974@whut.edu.cn (G.L.); leesonfirm@whut.edu.cn (Z.L.); 361037@whut.edu.com (S.S.); 375175@whut.edu.cn (R.C.); 2 Nanjing Zhongxing New Software Company, Nanjing 210012, China; sheng.jincheng@zte.com.cn; 3 School of Mechanical Engineering, Hubei University of Arts and Science, Xiangyang 441053, China; wzrvision@hbuas.edu.cn; 4 China Aerospace Science and Industry Corporation Second Research Institute, Beijing 100039, China; guansijia@azspace.cn

**Keywords:** quadruped robot, terrain perception, multi-signal fusion, support vector machine, parameter optimization

## Abstract

In unstructured environments, terrain perception is essential for stability and environmental awareness of Quadruped robot locomotion. Existing approaches primarily rely on visual or proprioceptive signals, but their effectiveness is limited under conditions of visual occlusion or ambiguous terrain features. To address this, this study proposes a multimodal terrain perception method that integrates acoustic features with proprioceptive signals. This terrain perception method collects environmental acoustic information through an externally mounted sound sensor, and combines the sound signal with proprioceptive sensor data from IMU and joint encoder of the quadruped robot. The method was deployed on the quadruped robot Lite2 platform developed by Deep Robotics, and experiments were conducted on four representative terrain types: concrete, gravel, sand, and carpet. Mel-spectrogram features are extracted from the acoustic signals and concatenated with the IMU and joint encoder to form feature vectors, which are subsequently fed into a support vector machine for terrain classification. For each terrain type, 400 s of data were collected. Experimental results show that the terrain classification accuracy reaches 78.28% without using acoustic signals, while increasing to 82.52% when acoustic features are incorporated. To further enhance the classification performance, this study performs a combined exploration of the SVM hyperparameters C and γ as well as the time-window length $w_{in}$. The final results demonstrate that the classification accuracy can be improved to as high as 99.53% across all four terrains.

## 1. Introduction

In unstructured environments, achieving accurate terrain perception is essential for the stable locomotion of quadruped robots. Currently, terrain is primarily inferred from the proprioceptive signals or externally mounted sensors of robots, including IMUs, LiDAR, and cameras. However, under poor illumination or in complex terrain, the perception results of a single sensor are prone to significant bias or even complete failure. To enhance the robustness of robotic operation, an increasing number of researchers have begun to shift their focus toward multimodal fusion frameworks that integrate and exploit complementary information from multiple sensing modalities.

When a quadruped robot traverses different types of terrain, the force profiles at its joints exhibit distinct patterns. Consequently, many researchers have focused on exploiting force sensors for terrain classification. Walas et al. [1] extracted time domain and frequency domain features from ankle force measurements of a humanoid robot and employed an SVM classifier to recognize five terrain types. Using FFT-based features, they reported a mean precision of 91.01 ± 1.94% and a mean recall of 90.75 ± 1.94%. Michal Bednarek and his team [2,3] introduced the HAPTR (Haptic Transformer) framework, which pioneered the use of Transformer architectures for tactile terrain classification in quadrupedal robots. Their method achieved a classification accuracy of up to 91.7% on an eight-class real-terrain dataset while reducing the CPU inference time per sample to less than 6 ms. Building on this work, their team subsequently proposed an improved variant, HAPTR2, which adopts a lightweight Transformer architecture together with multi-scale feature aggregation. HAPTR2 further reduces the per-sample inference time to 1.75 ms without sacrificing classification accuracy. Bednarek et al. [4,5] collected 8640 gait samples across six terrains using six-axis force sensors mounted on the Anymal robot, constructing an RNN model that achieved 93.59% classification accuracy. Ding et al. [6] tested foot-end normal and tangential force data across 12 terrains using the hexapod robot EISpider. Compared to manual feature extraction and CNN-based methods, their SVM classifier achieved 97.50% accuracy on 10 terrains and 85.83% accuracy on all 12 terrains.

Numerous researchers have also combined force sensors with additional signals to conduct terrain classification. Matej Hoffmann et al. [7] used the Puppy quadruped robot to collect joint encoder readings and foot pressure measurements while it traversed four distinct surfaces (plastic foil, cardboard, foam, and rubber) under five different gaits. Naive Bayes and SVM classifiers were then employed to assess how gait variations affect terrain classification performance. Yue et al. [8] employed IMU measurements, joint encoder readings, and torque sensor data to estimate real-time foot contact forces, and subsequently carried out online terrain classification using an SVM classifier. Experiments conducted on three terrain types yielded a classification accuracy of 96.7%. Csaba et al. [9] fused data from infrared (IR) distance sensors, motor force sensors, accelerometers, and ground contact force sensors to distinguish six indoor surface types. Employing a random forest model as the classifier, they achieved 96.2% accuracy in indoor terrain experiments and maintained 94% accuracy when testing the model on unseen data. Murphy et al. [10] integrated foot-end force measurements with foot-end sink depth into a unified feature representation, and achieved a terrain classification accuracy of 95% over four terrain types. Hendrik et al. [11] conducted experiments in which a single leg of the robot followed a predefined impact trajectory, while the remaining three legs maintained the robot in an upright stance. Accelerometer and force sensor data were then exploited to train an SVM-based terrain classification model, yielding an overall classification accuracy of 98%. Cheng et al. [12] constructed a 5-layer MLP deep neural network trained with vibration data collected across five terrain types. Tested at multiple speeds, terrain recognition accuracy reached 88.3% at 0.4 m/s, significantly outperforming traditional BP neural networks.

In addition, many researchers have opted not to use conventional force sensors, but instead exploit alternative sensory modalities to achieve non-contact terrain classification. R. Manduchi et al. [13] proposed a color-based terrain classification system capable of labeling detected obstacles as different terrain categories (e.g., grass, soil, rocks) based on color. Li et al. [14] proposed a path-following C-Terrain framework that leverages a ToF camera and a laser sensor to construct terrain zones along the robot’s trajectory. By extracting elevation-difference features from the acquired range data and training regression-based learning models, their method enables both terrain classification and terrain prediction in path-related regions. Paul et al. [15] developed a monocular camera-based terrain classification approach. They introduced a gradient descent-based heuristic to adaptively adjust the SURF Hessian threshold, thereby improving both the robustness and efficiency of feature extraction.

Extensive research has also been conducted on robot proprioceptive sensing and on approaches that combine these internal sensors with other sensing modalities. Lu et al. [16] proposed a terrain classification framework that integrates GRU-based temporal modeling with a semi-supervised learning strategy, achieving a terrain recognition accuracy of 91.4% while requiring labeled annotations for only 25% of the IMU data. Sophie et al. [17] proposed a terrain classification approach that relies exclusively on robot body signals and employs an integrated pipeline of energy-based feature computation, mRMR feature selection, PCA dimensionality reduction, and KNN classification. On three terrain types, their method achieved 97% accuracy in offline evaluation and 87% accuracy in online experiments. Clemens et al. [18] proposed a CNN-based terrain recognition method for humanoid robots that exploits 12-channel proprioceptive signals per footstep, comprising IMU measurements and joint current data. Using these multimodal internal signals, their approach achieved an average classification accuracy of 91.53% across five terrain types. Qin et al. [19] proposed an online terrain classification framework for quadrupedal robots based on acoustic signals. Extracting MFCC + Delta features and utilizing GMM for online classification, they achieved a 92.7% recognition rate across three typical terrains. Yu Chen et al. [20] proposed a CNN-based visual–proprioceptive fusion approach for terrain classification in UGVs, in which camera images are combined with proprioceptive measurements from onboard IMUs and joint encoders. Using this multi-sensor fusion framework, their method achieved an accuracy of 89.54% on datasets involving arbitrary vehicle motions.

For quadruped robots, achieving stable locomotion is inherently contingent on accurately recognizing the type and properties of the supporting terrain. Existing studies predominantly rely on either internal states or individual external sensors to perform terrain perception. However, any single source of information is subject to inherent limitations and vulnerability to noise or degradation. As a result, an increasing body of work has begun to focus on multi-signal fusion in order to enhance robustness and generalization across complex environments. Building on this line of research, we propose a terrain classification framework that integrates three signal sources, namely IMU measurements, joint encoder data, and foot-end acoustic signals. Specifically, IMU and encoder measurements capture terrain-dependent variations in the robot’s body motion and joint kinematics, whereas acoustic signals provide complementary cues from foot–ground contact interactions, reflecting intrinsic material and surface properties that are not fully observable from proprioception alone. Moreover, to improve classification performance and to provide practical guidance for parameter selection, we conduct extensive experiments over a range of SVM hyperparameters (C and γ) as well as temporal window lengths ($w_{in}$), and analyze their effects on recognition accuracy and robustness.

## 2. Methodology

### 2.1. Algorithm Framework

**Figure 1:**
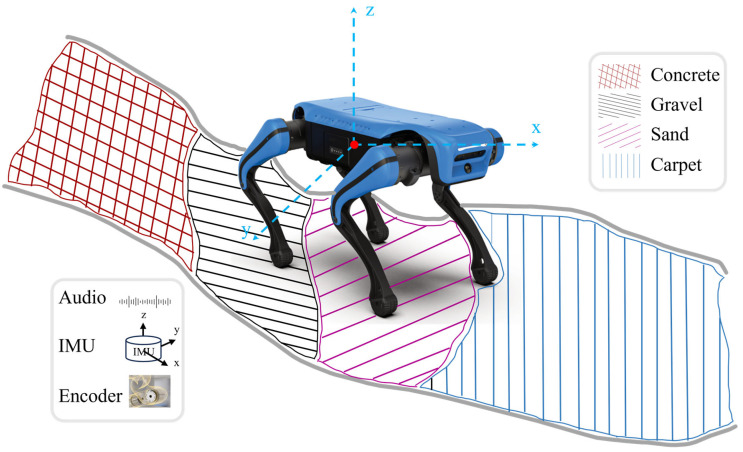
Experimental setup of the quadruped robot on four terrain types (concrete, gravel, sand and carpet) with simultaneous acquisition of audio, IMU and joint encoder signals.

The proposed multimodal terrain perception framework fusing acoustic signals consists of three key processes. The first step is data collection and preprocessing. The second is training the SVM classifier and obtaining the initial classification results. The last step involves optimizing the model parameters and generating the final classification outputs. The experimental platform used in this paper is the Lite2 quadruped robot manufactured by Deep Robotics, which has 12 degrees of freedom and supports multiple gait patterns with good terrain traversal capability. The perception system is equipped with various onboard sensors, and the main computing unit is an ARM-based GPU processor that can be accessed via remote desktop software for visualization. For convenient development, the robot also provides USB interfaces for external devices. As illustrated in Figure 1, signals were collected on four representative terrain types: concrete, gravel, sand, and carpet.

**Figure 2:**
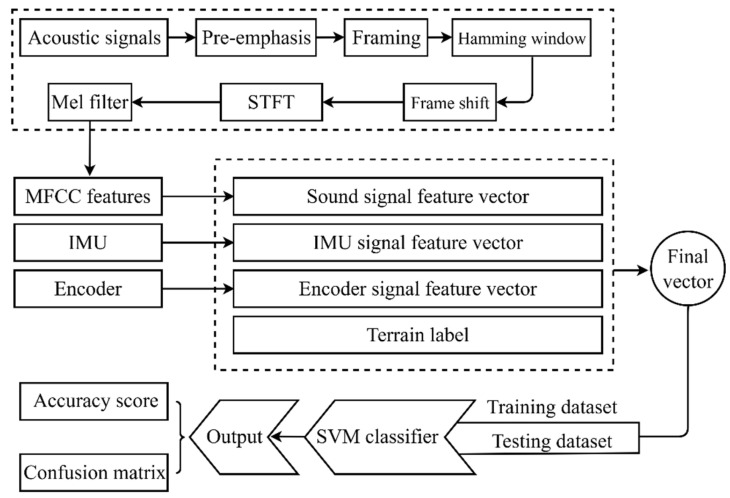
Overall pipeline of the proposed multimodal terrain perception method.

After the data was collected, the SVM terrain perception model was performed. As shown in Figure 2, to obtain the feature vectors required for model training, Mel-spectrogram features are first extracted from the raw audio recordings. The acoustic feature vectors are then concatenated with those derived from the IMU and joint encoder signals and finally associated with the corresponding terrain labels. At last, after constructing the complete set of feature vectors, the data are partitioned into training and test subsets to evaluate the classification performance of the proposed model.

Model performance is quantified using the confusion matrix as well as standard evaluation metrics, including overall classification accuracy, precision, recall, and *F*1-score. The formulas are shown as follows:

(1) $$\mathrm{Accuracy}\;=\frac{\mathrm{TP}+\mathrm{TN}}{\mathrm{TP}+\mathrm{TN}+\mathrm{FP}+\mathrm{FN}}$$

(2) $$Precision=\frac{\mathrm{TP}}{\mathrm{TP}+\mathrm{FP}}$$

(3) $$Recall=\frac{\mathrm{TP}}{\mathrm{TP}+\mathrm{FN}}$$

(4) $$F1-score=2\times \frac{Precision\times Recall}{Precision+Recall}$$

In the formulas above, TP, TN, FP, and FN are defined as:

TP (True Positives): The number of instances that are correctly predicted as positive.

TN (True Negatives): The number of instances that are correctly predicted as negative.

FP (False Positives): The number of instances that are incorrectly predicted as positive.

FN (False Negatives): The number of instances that are incorrectly predicted as negative.

Accuracy is the proportion of all evaluated samples that are classified correctly. Precision measures how reliable the positive predictions are for a given class; that is, among all samples predicted as that class, the fraction that truly belongs to it. Recall measures how completely the model identifies a given class; that is, among all samples that truly belong to that class, the fraction that is correctly predicted. The F1-score is the harmonic mean of precision and recall, providing a single value that balances the two, especially when class distributions are imbalanced.

### 2.2. Data Acquisition

**Figure 3:**
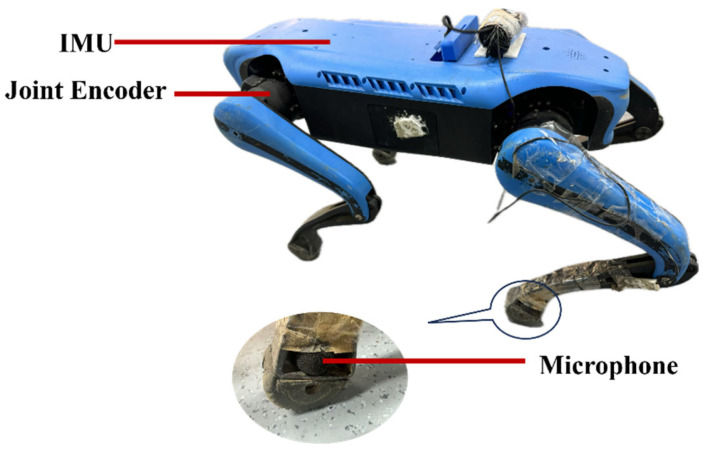
Mounting position of the external microphone on the quadruped robot.

To avoid interference from different locomotion patterns, all experiments in this paper are conducted under a single fixed gait. To maximally capture the acoustic signals generated by foot–ground impacts, a high-precision microphone is mounted at the foot end of the robot, as shown in Figure 3. The raw audio stream is transmitted via Ethernet from the robot to an external computer for processing. Through a remote desktop connection, we accessed the onboard perception computer of the robot and acquired the corresponding IMU and joint encoder signals. Owing to the limited computational resources of the onboard computer, the large volume of data, and the need to ensure data integrity so as to improve the robustness and accuracy of the terrain perception model, the robot is used solely as a data acquisition platform in this work. In future deployment, the trained model can be migrated to the onboard computer for edge execution according to task real-time and performance requirements.

Table 1 summarizes the three input modalities used for terrain perception. IMU and joint encoder signals are published as ROS topics at 50 Hz, whereas the acoustic signal is acquired by a USB microphone. The audio stream is published by a custom ROS node and recorded under the same time sequence as the internal sensor topics, with a sampling rate of 8 kHz. For each terrain, 400 s of synchronized data are collected, yielding 20,000 IMU and encoder samples and 3,200,000 audio samples, which are subsequently used for feature extraction and model training.

### 2.3. Signal Processing

Since the IMU and joint encoder signals are centrally managed by the motion-control computer and uploaded in a synchronized manner, these two data streams share a common system clock during acquisition, allowing their timestamps to be matched one-to-one. In contrast, the acoustic signal is independently acquired by an external microphone, whose sampling rate and time base differ from those of the internal system. To achieve temporal alignment among the three modalities (IMU, joint encoder, and audio), a unified time-synchronization scheme is implemented within the ROS framework: the microphone audio stream is integrated into ROS, and the audio acquisition node attaches system timestamps to the audio data so that they are consistent with the publication times of the internal sensor topics.

**Figure 4:**
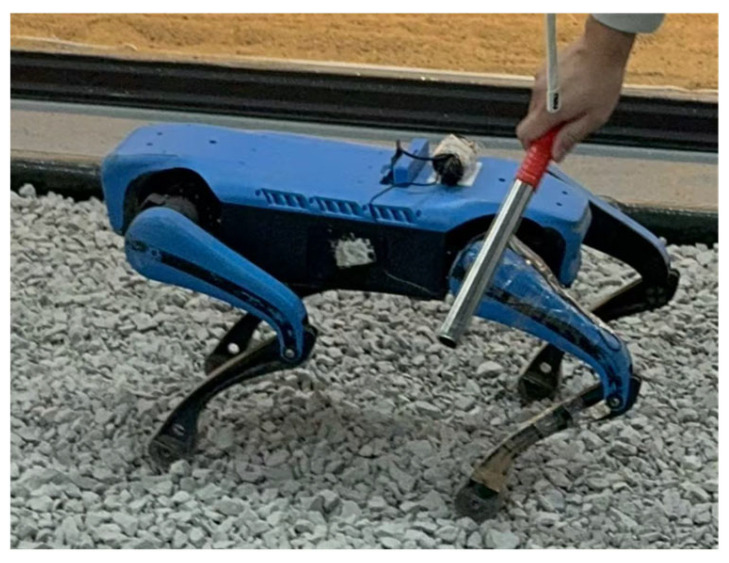
Manually induced impact on the quadruped robot used as a reference point for signal recording.

Since the robot begins publishing data from its initial prone posture, a common temporal reference is required to mark the onset of valid measurements. Once the robot has firmly stepped onto the four terrains, a manually induced impact is applied to the robot along the y-axis of motion, as shown in Figure 4.

**Figure 5:**
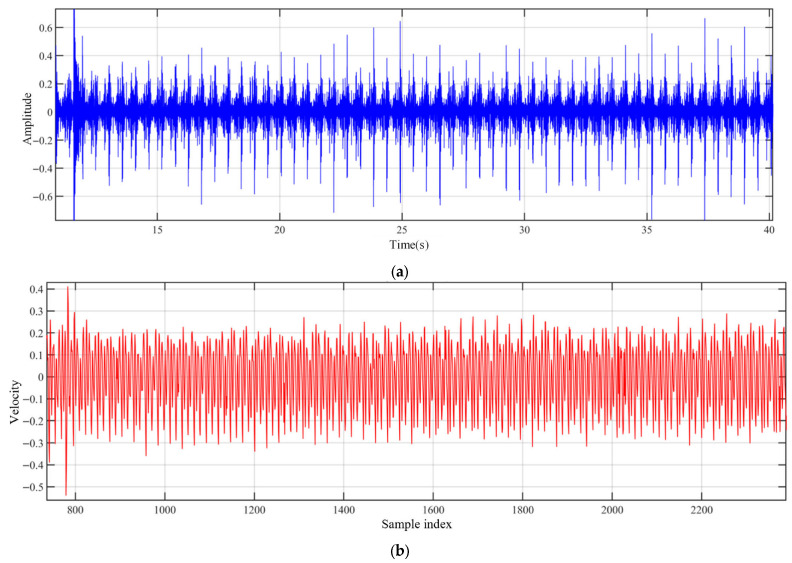
Signal perturbations induced by the impact event. (**a**) audio signal amplitude from sand terrain. (**b**) y-axis angular velocity of the robot.

This intentional impact induces a distinct transient in all three signal streams, which is subsequently used as the starting point for the usable data segment. As shown in Figure 5, the impact event appears as a pronounced discontinuity in the y-axis velocity measured by the onboard IMU and as a distinct impulsive signature in the acoustic signal. Furthermore, to avoid contamination of the terrain-recognition samples by the transient mechanical vibrations and acoustic reverberations induced by the impact, and to ensure that the robot has returned to a steady gait, only data starting 5 s after this synchronization event are used in this study.

**Figure 6:**
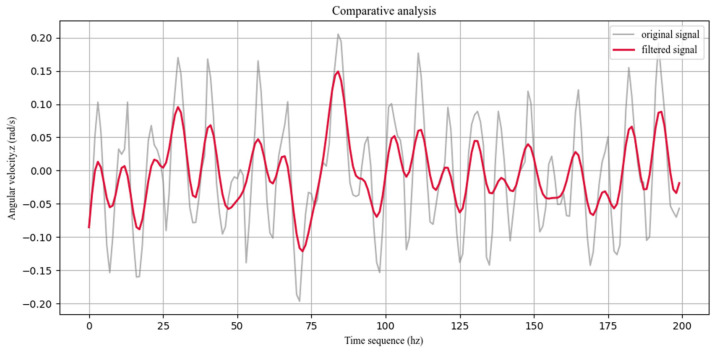
Comparison between original and low-pass filtered z-axis velocity signals of the robot.

To reduce random noise and high-frequency jitter in the raw IMU and joint encoder signals, a low-pass filtering stage is applied as a preprocessing step. The filter operates while preserving the original signal length and timestamps, effectively performing weighted smoothing on each sample so that the resulting trajectories more closely follow the underlying physical motion. As an illustrative example, the raw z-axis velocity of the robot during locomotion on concrete terrain exhibits pronounced high-frequency fluctuations and transient spikes, mainly caused by motor vibrations and internal sensor electrical noise. As shown in Figure 6, after processing the signal with a fourth-order Butterworth low-pass filter with a cutoff frequency of 5 Hz, these high-frequency components are effectively suppressed, and the overall waveform becomes substantially smoother.

For the audio signals, the original sampling frequency is 8 kHz, indicating that 8000 samples are recorded per second. To extract short-term spectral features, a 25 ms Hamming window with a 10 ms frame shift is employed for short-time Fourier transform (STFT) analysis and Mel-frequency cepstral coefficient (MFCC) feature extraction. This parameter configuration yields roughly 100 acoustic feature frames per second, which is sufficient to capture transient changes in sound during foot–terrain contact. To obtain a temporal resolution consistent with the proprioceptive signals of the robot, the acoustic features from two consecutive frames are averaged to form a 50 Hz acoustic feature sequence. In this way, the audio modality is synchronized with the IMU and joint signals on the time axis, enabling precise temporal alignment of the multimodal observations.

### 2.4. SVM Classification Model Development

To perform terrain recognition from multiple signal inputs, this study develops a terrain classification model based on a support vector machine (SVM). An SVM is a typical supervised learning method that can perform nonlinear classification in high-dimensional feature spaces, even when only limited training samples are available. Its core idea is to map the original inputs into a high-dimensional feature space via a kernel function and then determine the optimal separating hyperplane that maximizes the margin between classes. Compared with deep neural networks, SVMs have fewer trainable parameters, converge faster, and often exhibit stronger generalization in small-sample settings, making them well suited to the terrain recognition problem considered in this work, which fuses proprioceptive robot signals with external acoustic measurements.

After signal acquisition and preprocessing, features from different modalities are uniformly aligned and fused into a composite feature vector, as shown in the following equation:

(5) $$X_{t}=[X_{\mathrm{IMU},t},X_{\mathrm{Joint},t},X_{\mathrm{Audio},t}]\in {\mathbb{R}}^{31}$$

(6) $$y_{t}=\{0,1,2,3\}$$

Among these, $X_{\mathrm{IMU},t}$ represents the three-axis angular velocity and three-axis linear acceleration provided by the IMU, totaling 6 dimensions of features. $X_{\mathrm{Joint},t}$ denotes the joint angle information from the joint encoders of the four legs, totaling 12 dimensions. $X_{\mathrm{Audio},t}$ corresponds to the 13-dimensional features extracted from the audio signal via Mel-frequency cepstral coefficients (MFCC). The labels $y_{t}=\{0,1,2,3\}$ correspond to four test terrains, with each data point representing a fused 31-dimensional feature vector. The final feature vector $F_{t}$ is defined as follows:

(7) $$F_{t}=[X_{t},\;y_{t}]\in {\mathbb{R}}^{32}$$

To prevent overfitting caused by temporal leakage, this study divides the data into segments, separating the time-series data collected under different terrains into independent training and test sets. The division ratio is 8:2, with the training set used for model learning and the test set for model performance validation. The number of samples for each terrain is balanced to ensure the classifier has similar discriminative capabilities across different categories. To eliminate bias in the SVM classifier’s hyperplane solution caused by inconsistent feature scales, this study employs Z-score normalization before model training to remove dimensionality differences. The Z-score is defined as:

(8) $$x^{\prime }=\frac{x-\mu }{\sigma }$$

where μ and σ represent the mean and standard deviation of the feature, respectively. Model training was implemented in Python 3.12 using the scikit-learn library, employing a Radial Basis Function (RBF) kernel with the following kernel function form:

(9) $$K\left(x_{i},x_{j}\right)=\exp(-\gamma ||x_{i}-x_{j}|{|}^{2})$$

To obtain the optimal combination of model parameters, this study employs grid search and 5-fold cross-validation—common machine learning techniques—to systematically optimize the key hyperparameters C and γ of the support vector machine. Additionally, to further analyze the impact of time window parameters on model performance, comparative experiments were conducted under different Hamming window widths to explore the relationship between signal temporal resolution and classification accuracy.

The parameter C balances the trade-off between maximizing the classification margin and the misclassification penalty term. The parameter γ represents the bandwidth of the radial basis function (RBF) kernel, determining the influence range of samples in the feature space. The parameter $w_{in}$ denotes the width of the Hamming window, controlling the temporal resolution during the feature extraction stage. For each combination of parameters $\left(C,\gamma ,w_{in}\right)$, five-fold cross-validation was performed on the dataset, and the average classification accuracy was calculated as the model performance metric. Relevant experimental results and parameter sensitivity analysis will be discussed in detail in the Results section.

To comprehensively evaluate the performance of the established SVM terrain classification model, this study conducts quantitative analysis based on four core metrics: classification accuracy, precision, recall, and F1-score.

## 3. Results

### Experimental Setup

**Figure 7:**
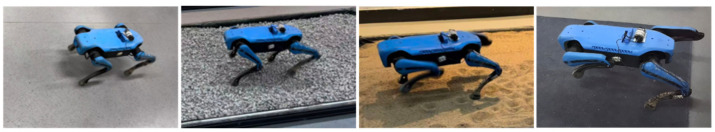
Experimental data collection on four terrains.

To minimize environmental noise interference, four terrain types were constructed in a sealed indoor environment: concrete, gravel, sand, and carpet, as shown in Figure 7. The robot traversed each terrain using a consistent diagonal walking gait. IMU and joint encoder data were recorded via remote connection to the perception host, while audio data was captured by microphones attached at the foot end.

For each terrain, 400 s of sensor data were collected, with IMU and joint encoder signals sampled at 50 Hz, yielding 20,000 data points per terrain. To avoid data leakage from future information during model training, we adopted a strict time-series partitioning scheme. The data were manually split into training and test sets in an 8:2 ratio according to temporal order, such that the training set contained only earlier samples and the test set contained only later samples. This strategy effectively prevents test data from leaking into the training process and ensures that the evaluation results reflect the true generalization performance of the model.

**Figure 8:**
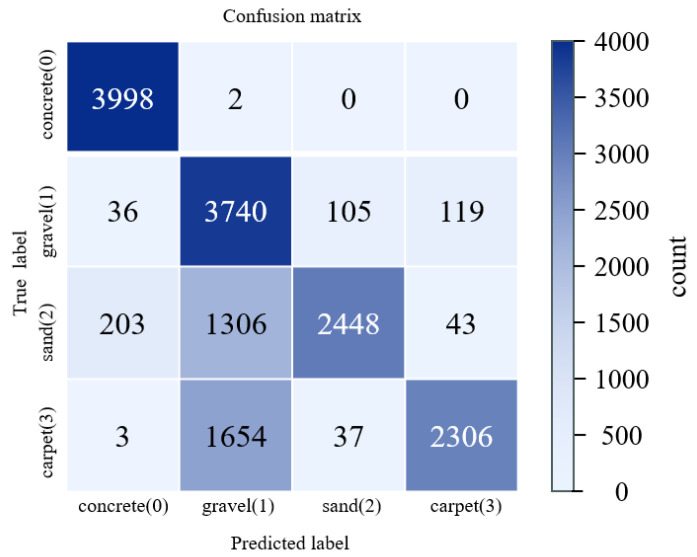
Confusion matrix of terrain classification using IMU and joint encoder data only.

As shown in Figure 8 and Table 2, the overall classification accuracy across the four terrain types is 78.28%. The highest accuracy is achieved on concrete surfaces, whereas misclassifications are particularly pronounced for gravel and sand. In addition, substantial confusion occurs between gravel and carpet. These errors likely arise from the predominantly flat experimental sites and from data being collected with the robot executing the same gait across terrains. Under such conditions, distinctive acoustic signal characteristics can provide complementary cues for terrain identification. We observe that during robot locomotion, foot–ground interactions generate markedly different vibration and sound patterns on different terrains: gravel surfaces produce strong high-frequency vibration noise, whereas sandy surfaces yield a softer, more muffled response.

**Figure 9:**
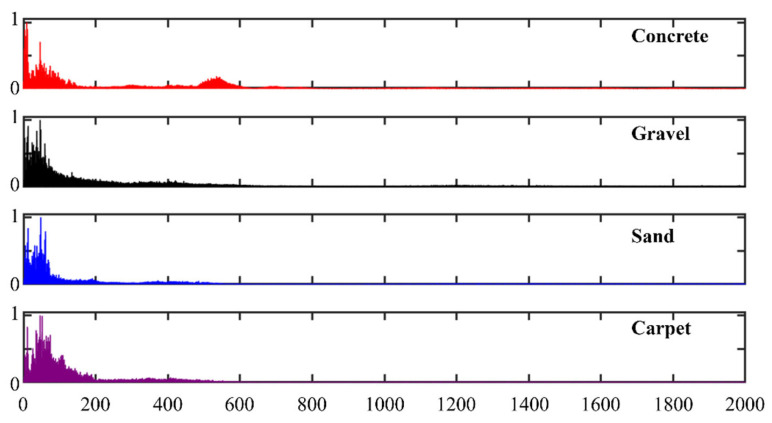
Frequency spectra of robot foot–ground contact sounds on four terrains (concrete, gravel, sand, and carpet).

As shown in Figure 9, the four terrain types exhibit distinct differences in their sound-frequency responses. For concrete, most spectral energy is concentrated below 1000 Hz, with the maximum amplitude occurring in the 0–50 Hz band. This indicates a strong interaction with low-frequency signals. The spectral energy gradually decreases as frequency increases, leading to a relatively smooth ground-vibration response. The spectrum for gravel also shows high energy at low frequencies, but its overall energy distribution is more uniform than that of concrete. Unlike concrete, which exhibits a pronounced dip followed by a rise in the mid-frequency range and thus maintains a secondary peak around 500 Hz, gravel does not show this resonance-like characteristic. Instead, its porous and irregular structure leads to rapid absorption and scattering-induced attenuation of the signal, together with weaker reflection at higher frequencies. Similarly to gravel, sand has an irregular surface, but its particles are much smaller and its very high porosity causes stronger absorption of incident sound. Around 0 Hz, the spectral amplitude for sand is markedly lower than that for gravel. For carpet, the spectrum reflects its superior sound-absorbing capability compared with the other three terrains: the energy of the sound waves remains relatively low over all frequencies, and the strong damping causes progressive energy loss during propagation. The waves are continuously scattered and absorbed by the soft, irregular carpet surface, resulting in a generally low-amplitude spectrum in which the portion above a given magnitude threshold occupies only a narrow frequency band.

To improve the performance of the SVM-based terrain classification model, we incorporate acoustic features as additional input signals. Although the original WAV recordings preserve the full waveform information, directly using the 8 kHz time-domain samples may introduce substantial redundancy and increase sensitivity to noise and minor temporal misalignments. Moreover, while the waveforms from different terrains can appear visually similar, their spectral characteristics differ more distinctly. Therefore, we employ the short-time Fourier transform (STFT) to obtain a time–frequency representation and extract spectral features.

**Figure 10:**
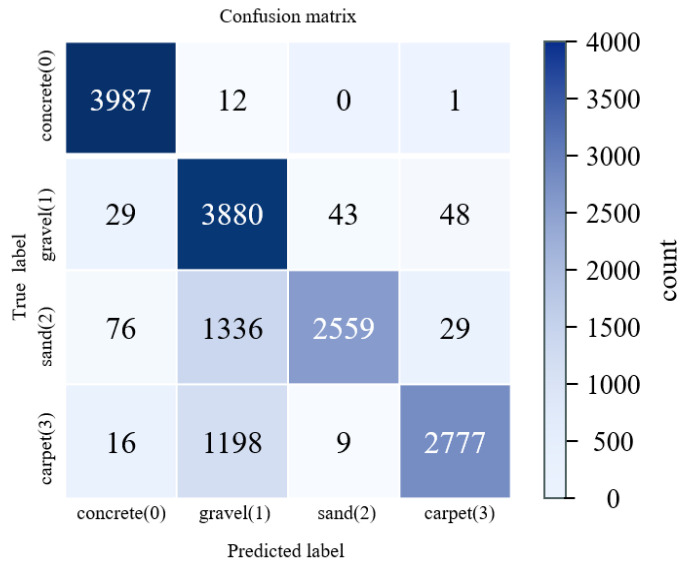
Confusion matrix of terrain classification using fused IMU, joint encoder, and audio features.

We then compute Mel-frequency cepstral coefficients (MFCCs) from the spectra. The use of the Mel filter bank is perceptually motivated. However, in our setting its main benefit is that it performs a non-uniform frequency aggregation that emphasizes low-frequency structures and yields a smoother approximation of the spectral envelope. This reduces sensitivity to small frequency shifts and high-frequency fluctuations that may arise from environmental disturbances or sensor noise. In addition, the logarithmic compression of Mel-band energies mitigates amplitude variability caused by changes in contact intensity and microphone gain, and the subsequent discrete cosine transform (DCT) decorrelates the log-Mel features and provides a compact representation. Overall, this MFCC pipeline reduces feature redundancy while preserving terrain-discriminative contact patterns, which contributes to improved robustness and generalization for robotic audio-based terrain classification. The extraction process is expressed as follows:

(10) $$c_{n}=\sum_{k=0}^{K-1}\log(M_{k})\cdot \cos[\frac{\pi n}{K}(k+\frac{1}{2})]$$

where $c_{n}$ is the n-th MFCC, K is the number of Mel filters, $log(M_{k})$ is the k-th Mel-band energy, and cos (⋅) denotes the cosine basis function of the DCT. The first 13 MFCCs are used as audio features. To obtain a suitable spectrum for analysis and ensure signal continuity, the audio signal is segmented into overlapping frames using a Hamming window. Each frame has a duration of 25 ms with a 10 ms frame shift, resulting in MFCC features at 100 Hz. To match the 50 Hz sampling rate of the IMU and joint encoder signals, every two consecutive MFCC frames are averaged, yielding audio MFCC features at 50 Hz. Following the same model-training procedure as described above, the entire dataset is split into training and test sets in an 8:2 ratio. The split is performed manually in temporal order to prevent temporal data leakage between the training and test sets. The resulting confusion matrix is shown below in Figure 10.

The confusion matrix illustrates the class-wise prediction results of the proposed multimodal model, showing the distribution of correct and incorrect classifications across terrain types. However, the model still struggles to distinguish between gravel (Class 1) and sand (Class 2). The detailed performance metrics are summarized in Table 3.

Both the figure and the table show that, although incorporating audio signals increases the overall classification accuracy from 78.28% to 82.52%, there is still considerable room for improvement in distinguishing gravel, sand, and carpet surfaces.

To optimize the model’s classification performance, we next evaluate multiple combinations of the two key SVM hyperparameters, C and γ. The SVM classifier is initially configured with C=1 and γ=0.1. Following the exponential grid-search strategy for C and γ proposed by Hsu et al. [21]., we explore two exponential levels above and below these initial values. The resulting search range is defined as follows:

(11) C∈{0.01,0.1,1,10,100},γ∈{0.001,0.01,0.1,1,10}

Additionally, we observed that the spectral differences among the four terrain types primarily appear in the low-to-mid frequency range. In our current SVM classification model, the Hamming window width is set to $w_{in}=0.04\;s$, whereas the robot’s step cycle during data acquisition is approximately 1 s. We hypothesize that a 0.04 s audio window may be too short to capture complete frequency-domain characteristics. Therefore, for the Hamming window width $w_{in}$, we designed ten experimental settings ranging from 0.1 s to 1 s. This configuration allows us to capture at least five frames and at most one full gait cycle of signal features, enabling us to examine their effect on the final classification accuracy. The window width settings are as follows:

(12) $$w_{in}\in \{0.1,0.2,0.3,0.4,0.5,0.6,0.7,0.8,0.9,1.0\}$$

**Figure 11:**
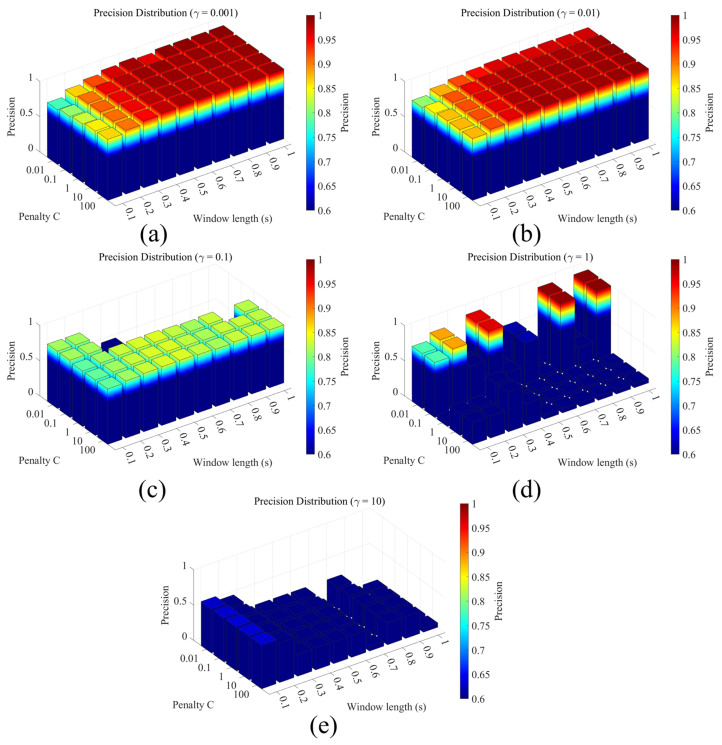
Precision distribution over SVM hyperparameters C, γ and $w_{in}.$ (**a**) γ=0.001; (**b**) γ=0.01; (**c**) γ=0.1; (**d**) γ=1; (**e**) γ=10.

After training the SVM terrain classification model with the above parameter combinations, we obtained the corresponding classification accuracy comparison figure, as shown below in Figure 11.

A comparison of the five plots shows that when the penalty factor C is small, the model is subject to stronger regularization constraints. The decision boundary becomes relatively smooth, but underfitting is more pronounced, leading to lower overall accuracy. As C increases, the model’s tolerance for training errors decreases. When C=1 or C=10, the classification accuracy improves. However, when C reaches 100, especially for γ>1, the model exhibits clear overfitting tendencies, and the accuracy decreases, as reflected by large fluctuations in the histogram heights.

For the parameter γ, when γ=0.001 or 0.01, the kernel function induces a relatively smooth mapping, forming a wide decision boundary in the feature space. This yields high overall accuracy and a stable distribution trend. Under these values, the accuracy differences among various combinations of C and window length $w_{in}$ are small, indicating strong model generalization. When γ exceeds 1, however, the histograms exhibit severe fluctuations, and the accuracy drops sharply for certain parameter combinations—particularly for short window lengths (0.1–0.3 s) and large C—revealing pronounced overfitting. This reduces generalization capability and can cause a pronounced performance drop. Therefore, under our experimental setting, smaller values of γ are generally preferred to obtain smoother and more robust classification results.

With respect to the time window $w_{in}$, as the window length increases from 0.1 s to 1.0 s, the model accuracy shows an overall increasing trend. Shorter windows (0.1–0.3 s) fail to adequately capture feature variations within a single gait cycle, resulting in insufficient temporal information in each input segment and reduced stability of the classification decisions. The specific accuracy values are listed in the following tables.

Table 4, Table 5, Table 6, Table 7 and Table 8 indicate that the system achieves its highest accuracy of 99.53% when the hyperparameters are set to C=0.01, γ=0.001, and a window length $w_{in}=1\;s$.

## 4. Discussion and Conclusions

This study systematically evaluated the recognition performance of an SVM-based terrain classification model under different parameter configurations using multimodal inputs from IMU measurements, joint encoder data, and acoustic signals. In contrast to approaches that perform terrain recognition using acoustic information alone, the proposed method does not treat audio as a standalone modality. Instead, we use foot end acoustic signals as complementary evidence to proprioceptive measurements, because acoustic cues can reflect contact characteristics associated with material properties and surface texture that may remain ambiguous when only kinematic information is available. From this perspective, the key contribution of our framework is the fusion of three signal sources and the resulting improvement in robustness under modality specific uncertainty, rather than a straightforward extension of prior acoustic approaches. Moreover, the present study evaluates performance under an offline setting with fixed temporal windows in order to systematically analyze the effects of temporal window length and SVM hyperparameters. Extending the framework to online terrain recognition with lower decision latency remains an important direction for future work.

It should be noted that, although placing the acoustic sensor near the foot helps capture contact related sound cues, the recordings may also include interference from robot induced sources such as structural vibrations, motor and transmission noise, and transient joint impacts. Future work will investigate noise-robust acquisition and processing strategies, including improved mechanical isolation, reference-based suppression using proprioceptive measurements, and extensions toward online recognition in more complex environments. Regarding our choice of MFCC features, it is important to note that the foot–ground interactions in our experiments cover both compliant and relatively rigid contacts, rather than being limited to bare rigid impacts. Accordingly, in future work we will systematically compare MFCCs with traditional (linear-frequency) cepstral features and other spectral representations to assess their suitability across different contact conditions.

The results indicate that, under the baseline setting, simply introducing acoustic features as an additional input modality improves the model’s overall classification accuracy from 78.28% to 82.52%, suggesting that audio provides complementary information beyond proprioception. When $w_{in}$ is increased to 1 s, the classifier can exploit richer contact related acoustic context, and the peak accuracy reaches 99.53%. However, this improvement comes with a trade-off, including increased decision latency and potentially higher sensitivity to acoustic disturbances, which may reduce robustness under practical variability. Future work will investigate adaptive windowing and modality balancing strategies to maintain high accuracy while improving robustness and real time applicability.

We found that the model performance is highly sensitive to the joint tuning of the kernel parameter γ, the penalty factor C, and the time-window length $w_{in}$. When γ is excessively large, the model overreacts to local noise, leading to degraded generalization capability. When C is too small, the model fails to adequately fit complex terrain patterns, and when $w_{\mathrm{in}}$ is too short, it cannot capture sufficient temporal information related to terrain dynamics. The experiments indicate that when γ∈[0.001,0.01], C∈[1,10], and $w_{in}\geq 0.6\;s$, the model achieves an effective balance between smooth decision boundaries and high classification accuracy. The highest accuracy of 99.53% is obtained with hyperparameters C=0.01, γ=0.001, and $w_{in}=1\;s$. Compared with traditional unimodal terrain recognition methods (e.g., vision-only or IMU-only approaches), the multimodal signal fusion model developed in this study achieves superior recognition accuracy across diverse terrain conditions.

This fusion approach offers several advantages. It requires only an external microphone and access to the robot’s ROS system to acquire diverse signals, including internal robot data. These signals are then fed into an SVM-based classification model to produce terrain labels, and further model optimization can be explored by tuning combinations of several hyperparameters. However, in this study the experimens were conducted with a fixed time-window configuration. Although this setting provides reasonable classification performance on most terrains, it may fail to capture key features during sudden obstacle encounters or frequent gait transitions. Future work could therefore introduce an adaptive window-length mechanism based on gait cycles, which remains an important direction for further investigation. Furthermore, although the proposed model has been validated on four representative terrains, it should be noted that all experiments were conducted in a controlled indoor setting under relatively ideal conditions with limited external disturbances. Accordingly, the reported performance primarily reflects the model’s effectiveness in indoor or similarly low-interference scenarios. In practical deployment, however, terrain perception must cope with substantially greater variability, including mixed-terrain transitions, heterogeneous road conditions, ambient noise, humidity variations, and speed fluctuations, which may induce domain shifts and degrade generalization performance. Future work will therefore extend the evaluation to outdoor and more challenging settings and to broader multi-terrain datasets, with the goal of more rigorously assessing robustness and generalization and facilitating reliable real-world deployment.

## Figures and Tables

**Table 1:** Specifications of the multimodal input signals (IMU, joint encoder, and audio).

Category	IMU	Joint Encoder	Audio
Sampling frequency (Hz)	50	50	8000
Acquisition device/system	ROS system	ROS system	External microphone (BOYA tech, Beijing, China)
Measured variables	Angular velocity (xyz), Linear acceleration (xyz)	Joint position (12 DOF)	Acoustic vibration signal
Sensor placement	Robot trunk	All 4 legs	Mounted near robot base
Data format	CSV	CSV	WAV
Data volume per terrain	20,000 samples	20,000 samples	3,200,000 samples
Purpose	Dynamic motion and posture analysis	Motion coordination and torque estimation	Terrain texture and impact detection

**Table 2:** Terrain Classification Accuracy Table Using IMU Data and Joint Encoder Data Only.

Terrain (Label)	Precision	Recall	F1-Score	Support
Concrete (0)	94.29%	99.95%	97.03%	4000
Gravel (1)	55.77%	93.50%	70.13%	4000
Sand (2)	94.52%	61.19%	74.26%	4000
Carpet (3)	93.44%	57.65%	71.36%	4000
Accuracy	—	—	78.28%	16,000

**Table 3:** Terrain Classification Accuracy Table Using Fused IMU, Joint Encoder, and Audio Features.

Terrain (Label)	Precision	Recall	F1-Score	Support
Concrete (0)	97.05%	99.68%	98.35%	4000
Gravel (1)	60.38%	97.00%	74.43%	4000
Sand (2)	98.01%	63.98%	77.42%	4000
Carpet (3)	97.27%	69.43%	81.02%	4000
Accuracy	-	-	82.52%	16,000

**Table 4:** Terrain Classification Accuracy Table (γ = 0.001).

Parameter Combination (γ = 0.001)	$w_{in}$ = 0.1	$w_{in}$ = 0.2	$w_{in}$ = 0.3	$w_{in}$ = 0.4	$w_{in}$ = 0.5	$w_{in}$ = 0.6	$w_{in}$ = 0.7	$w_{in}$ = 0.8	$w_{in}$ = 0.9	$w_{in}$ = 1.0
C = 0.01	77.64%	86.83%	92.16%	96.51%	98.52%	96.02%	99.37%	99.26%	99.32%	99.53%
C = 0.1	80.71%	90.62%	95.55%	97.90%	98.89%	99.09%	98.84%	99.03%	98.78%	98.92%
C = 1	82.86%	91.15%	95.52%	97.96%	98.90%	98.91%	98.44%	98.91%	98.65%	99.07%
C = 10	85.62%	90.63%	95.36%	97.77%	98.50%	98.65%	98.44%	98.68%	98.65%	98.92%
C = 100	86.55%	90.44%	94.86%	97.31%	98.43%	98.65%	98.15%	98.57%	98.52%	98.92%

**Table 5:** Terrain Classification Accuracy Table (γ = 0.01).

Parameter Combination (γ = 0.01)	$w_{in}$ = 0.1	$w_{in}$ = 0.2	$w_{in}$ = 0.3	$w_{in}$ = 0.4	$w_{in}$ = 0.5	$w_{in}$ = 0.6	$w_{in}$ = 0.7	$w_{in}$ = 0.8	$w_{in}$ = 0.9	$w_{in}$ = 1.0
C = 0.01	80.76%	88.06%	92.12%	94.57%	96.34%	96.70%	96.37%	96.83%	96.56%	96.62%
C = 0.1	85.18%	91.74%	95.55%	97.54%	98.21%	98.16%	98.28%	98.47%	98.07%	98.81%
C = 1	87.41%	91.93%	96.10%	98.19%	98.77%	98.57%	98.75%	98.81%	98.78%	99.24%
C = 10	87.68%	91.96%	95.83%	98.14%	98.77%	98.57%	98.65%	98.70%	98.91%	99.24%
C = 100	87.67%	91.96%	95.83%	98.14%	98.77%	98.57%	98.65%	98.70%	98.91%	99.24%

**Table 6:** Terrain Classification Accuracy Table (γ = 0.1).

Parameter Combination (γ = 0.1)	$w_{in}$ = 0.1	$w_{in}$ = 0.2	$w_{in}$ = 0.3	$w_{in}$ = 0.4	$w_{in}$ = 0.5	$w_{in}$ = 0.6	$w_{in}$ = 0.7	$w_{in}$ = 0.8	$w_{in}$ = 0.9	$w_{in}$ = 1.0
C = 0.01	81.26%	81.27%	31.26%	56.29%	31.30%	6.24%	6.26%	31.28%	31.29%	6.26%
C = 0.1	81.26%	81.27%	31.26%	56.29%	31.30%	6.24%	6.26%	31.28%	31.29%	6.26%
C = 1	78.60%	81.14%	82.74%	83.35%	83.56%	82.36%	81.39%	82.73%	57.67%	82.18%
C = 10	78.78%	81.17%	82.77%	83.40%	83.57%	82.41%	81.44%	82.75%	82.75%	82.22%
C = 100	78.78%	81.17%	82.77%	83.40%	83.57%	82.41%	81.44%	82.75%	82.75%	82.22%

**Table 7:** Terrain Classification Accuracy Table (γ = 1).

Parameter Combination (γ = 1)	$w_{in}$ = 0.1	$w_{in}$ = 0.2	$w_{in}$ = 0.3	$w_{in}$ = 0.4	$w_{in}$ = 0.5	$w_{in}$ = 0.6	$w_{in}$ = 0.7	$w_{in}$ = 0.8	$w_{in}$ = 0.9	$w_{in}$ = 1.0
C = 0.01	78.06%	88.47%	54.52%	96.18%	27.90%	61.63%	6.26%	99.25%	28.03%	99.52%
C = 0.1	78.06%	88.47%	54.52%	96.18%	27.90%	61.63%	6.26%	99.25%	28.03%	99.52%
C = 1	6.25%	6.25%	6.25%	6.25%	6.25%	6.24%	6.26%	6.26%	6.24%	6.26%
C = 10	31.25%	31.26%	56.26%	31.27%	6.25%	6.24%	6.26%	6.26%	6.24%	6.26%
C = 100	31.25%	31.26%	56.26%	31.27%	6.25%	6.24%	6.26%	6.26%	6.24%	6.26%

**Table 8:** Terrain Classification Accuracy Table (γ = 10).

Parameter Combination (γ = 10)	$w_{in}$ = 0.1	$w_{in}$ = 0.2	$w_{in}$ = 0.3	$w_{in}$ = 0.4	$w_{in}$ = 0.5	$w_{in}$ = 0.6	$w_{in}$ = 0.7	$w_{in}$ = 0.8	$w_{in}$ = 0.9	$w_{in}$ = 1.0
C = 0.01	62.90%	56.29%	31.31%	31.26%	31.24%	31.23%	6.26%	31.23%	6.21%	6.22%
C = 0.1	63.11%	56.30%	31.31%	31.26%	31.25%	31.23%	6.26%	31.23%	6.21%	6.22%
C = 1	63.24%	56.30%	31.31%	31.26%	31.25%	31.23%	6.26%	31.23%	31.22%	6.22%
C = 10	63.24%	56.30%	31.31%	31.26%	31.25%	31.23%	6.26%	31.23%	31.22%	6.22%
C = 100	63.24%	56.30%	31.31%	31.26%	31.25%	31.23%	6.26%	31.23%	31.22%	6.22%

## Data Availability

The original contributions presented in this study are included in the article. Further inquiries can be directed to the corresponding author.
